# Innovations in Band and Loop Space Maintainers: Transition From Conventional to Advanced

**DOI:** 10.7759/cureus.85234

**Published:** 2025-06-02

**Authors:** Mridula Goswami, Vashi Narula, Rimshheanam Rimshheanam

**Affiliations:** 1 Pediatric and Preventive Dentistry, Maulana Azad Institute of Dental Sciences, New Delhi, IND

**Keywords:** clinical pediatric dentistry, interceptive orthodontics, premature tooth loss, primary molar teeth, space maintainer

## Abstract

Primary dentition helps jaw development, mastication, speech, and esthetics and guides and maintains space for the permanent successors until they erupt. Premature tooth loss due to dental trauma and caries is an emerging concern in pediatric patients, necessitating space maintenance to prevent loss of arch length and maintain space for developing permanent teeth. Innovations in conventional space maintainers have significantly improved patient and operator comfort by reducing chairside time and increasing durability and operative efficacy. This case series is a compilation of five cases of young children in the age group of five to seven years with non-restorable primary molars requiring extraction. Clinical examination, radiographic assessment, and space analysis indicated the need for space management in each case. Different modified and innovative types of band and loop space maintainers were customized to the needs of each patient. Six-month follow-up showed satisfactory space maintenance and patient comfort in three cases. Early intervention with space maintainers customized to each case was found crucial for the prevention of arch length loss and malocclusion. Modifications based on individual patient requirements significantly improved success, patient compliance, and comfort.

## Introduction

Primary dentition plays a crucial role in a child's maxillofacial development, impacting speech, mastication, and appearance. It also helps in preventing harmful oral habits and guides the eruption and alignment of permanent teeth [1]. Premature tooth extraction before the eruption of a permanent successor can cause loss of arch length, leading to delayed or ectopic eruption, impaction, or worsened malocclusion. Hence, the use of an efficient, durable, and feasible space maintainer is recommended.

According to Boucher, a space maintainer is defined as a fixed or removable appliance designed to preserve the space created by the premature loss of a primary tooth or group of teeth [2]. Band and loop is the most commonly used fixed space maintainer in pediatric dentistry for premature loss of a single tooth. The conventional band and loop space maintainer is easy to construct, durable, and economical. However, there are several disadvantages associated with this appliance, such as the need for an accurate dental impression, additional laboratory procedures, multiple visits, the possibility of a metal allergy, and lack of esthetics [3]. To overcome these limitations, several advancements have been made to increase patients' comfort, reduce fabrication time, and improve esthetics. The present case series is a compilation of different types of band and loop space maintainers customized to the requirements of each case.

## Case presentation

This case series reports five young patients in the age group of five to seven years who visited the Department of Pediatric and Preventive Dentistry with irreversibly damaged primary molars requiring extraction, followed by space maintenance. For each case, a comprehensive demographic, medical, and dental history was obtained, followed by informed consent from the parents/guardians. A pre-treatment clinical and radiographic evaluation was performed. Based on the space analysis, an appropriate space maintainer was selected. The indicated tooth was extracted under local anesthesia, followed by the fabrication and insertion of the space maintainer. Basic non-pharmacological behavior management techniques such as Tell-Show-Do, positive reinforcement, and distraction were employed during the adaptation, isolation, and cementation of the space maintainers in each case. These approaches helped gain the child's cooperation during the procedure. Postoperative radiographs were taken to assess the placement, and regular follow-up appointments were scheduled (24 hours, one week, and one, three, and six months) to monitor the function and integrity of the space maintainer.

Case 1: Preformed band and custom-made loop space maintainer

Case Description

A six-year-old female patient had pain in the lower left back tooth region for one month. On intraoral examination, the root stump in relation to 75 and dental caries with 74 were observed. Radiovisiography (RVG) of tooth 75 showed crown loss with furcal radiolucency, requiring extraction. The space discrepancy was found to be 0.5 mm (< 2 mm) using Tanaka and Johnston space analysis; hence, the use of a space maintainer was indicated [4]. As the permanent lower central incisors had partially erupted and there was a unilateral space discrepancy of 0.5 mm, a band and loop space maintainer was preferred over a lingual arch.

Treatment Plan

**Figure 1:**
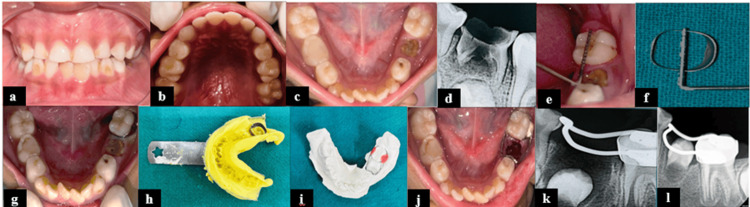
Preformed band and custom-made loop space maintainer for Case 1. (a) Intraoral frontal view; (b) intraoral preoperative maxillary occlusal view; (c) intraoral preoperative mandibular occlusal view showing root stump in relation to 75 and dental caries with 74; (d) radiograph showing crown destruction and root resorption involving furcation in relation to 75; (e) assessing the mesiodistal diameter of tooth 36; (f) assessing the mesiodistal diameter of the band; (g) assessment of band fit; (h) alginate impression with band; (i) working model with band and loop; (j) band and loop cementation with respect to 75; (k) radiograph showing band and loop with respect to 75; and (l) follow-up radiograph at six months.

A pre-formed band was desired for the case to improve patient comfort and reduce chairside time, as the child was very young. The smallest well-fitted preformed band of appropriate size (38, mPEDO, Mumbai, India) was adapted on 36, followed by a full arch impression using alginate impression material. At the second appointment, cementation of the space maintainer was done after extraction using type-I GIC (GC Fuji I Glass Ionomer Luting Cement Set, GC International AG, Lucerne, Switzerland), and postoperative instructions were given (Figure 1). Assessments at 24 hours, one week, and one, three, and six months after showed satisfactory results [5].

Case 2: Prefabricated band and loop space maintainer

Case Description

A seven-year-old male patient reported pain in the lower right back tooth region for one month. Clinical evaluation showed proximal caries in relation to 74 and 84. RVG evaluation showed root resorption with respect to tooth 84, which was planned for extraction. Tanaka and Johnston's space analysis showed a space discrepancy of 1.5 mm, indicating space maintainer fabrication [4]. As the child showed negative behavior on Frankl's Behavior Rating Scale (FBRS-2), a prefabricated band and loop space maintainer was chosen to reduce the operatory steps and increase patient cooperation. The space maintainer was cemented after extraction in a single appointment.

Appliance Design

**Figure 2:**
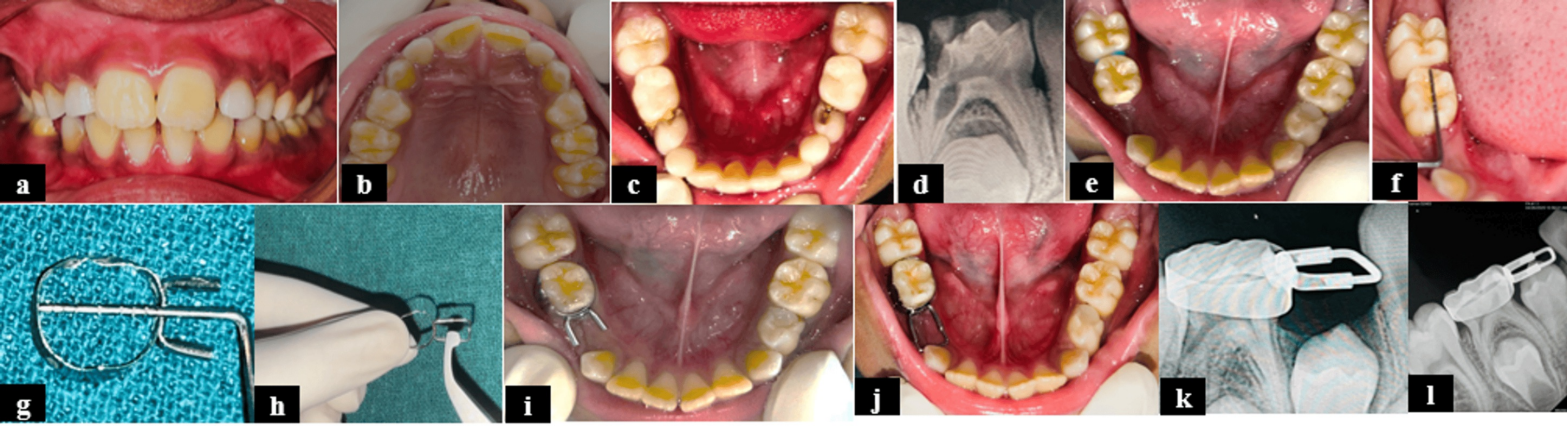
Prefabricated band and loop space maintainer for Case 2. (a) Intraoral frontal view; (b) intraoral preoperative maxillary occlusal view; (c) intraoral preoperative mandibular occlusal view showing proximal caries in relation to 74 and 84; (d) radiograph showing caries with internal root resorption with tooth 84; (e) mandibular occlusal view after mouth preparation; (f) assessment of mesiodistal diameter of tooth 85; (g) assessment of mesiodistal diameter of the band; (h) placement of the band inside the tube by using a Howe pliers; (i) checking the fit of the band; (j) preformed band and loop cementation with respect to 84; (k) radiograph showing band and loop with respect to 84; and (l) follow-up radiograph at six months.

An appropriate size of prefabricated band (35, mPEDO, Mumbai, India) was adapted onto tooth 85. A straight loop was adjusted as per the mesiodistal dimensions of the lost tooth. The parallelism between the two ends of the loop and the band's tube was ensured, and the loop was placed inside the band's tube using Howe pliers. Post-assessment, the tubes were crimped. Assessments at 24 hours, one week, and one, three, and six months after showed satisfactory results (Figure 2) [5].

Case 3: Glass fiber-reinforced composite (GFRC) space maintainer

Case Description

A seven-year-old female patient came with pain in the lower left back tooth region for one week. Clinical and radiographic evaluation showed grossly decayed 74 with external root resorption and furcation radiolucency. The space discrepancy was found to be 1 mm using Huckaba's space analysis [4]. The parents of the patient were esthetically concerned, as the girl was going to school and wanted a space maintainer with low visibility. Therefore, a metal-free GFRC space maintainer was planned for the child. At the first visit, tooth 74 was extracted, followed by GFRC loop cementation in the next visit.

Appliance Design

**Figure 3:**
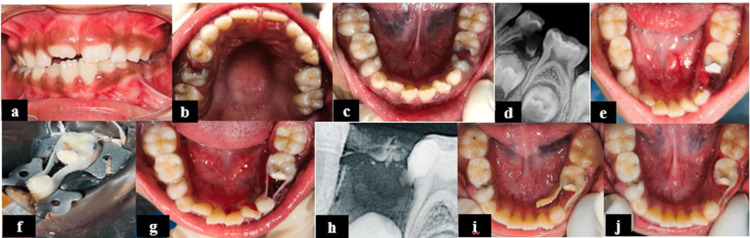
Glass fiber-reinforced composite (GFRC) space maintainer for Case 3. (a) Intraoral frontal view; (b) intraoral preoperative maxillary occlusal view; (c) intraoral preoperative mandibular occlusal view showing grossly decayed 74 and proximal caries with tooth 75; (d) radiograph showing non-restorable carious teeth with respect to 74 with furcal involvement; (e) mandibular occlusal view after mouth preparation; (f) glass fiber-reinforced composite (GFRC) space maintainer placement under rubber dam isolation; (g) GFRC space maintainer on teeth 73 and 75; (h) RVG of teeth 73, 74, and 75 region after GFRC placement; (i) failure of GFRC due to debonding at one-month follow-up; (j) follow-up at six months.

A 2 mm thick glass fiber splint (Kids-e-Dental E-Splint, Mumbai, India) was cut to the appropriate length and adapted on the buccal and lingual sides of 73 and 75. The teeth were etched with 35% orthophosphoric acid for 20 seconds, rinsed for 15 seconds, air-dried for 10-15 seconds, wetted with a bonding agent (Ivoclar Vivadent Te-Econom, 5th Generation Bonding Agent, Gurugram, India), and light-cured for 40 seconds. A thin layer of flowable composite (Ivoclar Vivadent Te-Econom Flow Refills Flowable Composite Resin) was applied on the treated surfaces to reinforce the splint. Gingival clearance and occlusal interference were checked, followed by finishing using composite finishing burs. Finally, the bonding agent was applied over the fiber frame and light-cured for 40 seconds. The one-month follow-up showed failure due to debonding at the enamel-composite interface from the lingual aspect (Figure 3). Assessment criteria by Kirzioglu Z (2004) [6].

Case 4: Ceramic band and loop space maintainer

Case Description

A five-year-old male patient reported pain in the lower left back tooth for one month. Intraoral examination showed grossly decayed 74 with Grade II mobility and restored 84. RVG evaluation revealed root resorption with furcal involvement with tooth 74, which was planned for extraction. The space discrepancy was found to be 1 mm using the Boston University Approach [7]. The patient's parent wanted a well-fitted esthetic space maintainer with very little chairside time, as the child was very small. A metal-free ceramic band and loop space maintainer was planned for this case to eliminate operator steps and enhance esthetics. At the first appointment, extraction was performed in relation to 74. To minimize the risk of space loss, the patient was recalled within one week post-extraction. As soon as satisfactory socket healing was observed, impressions were taken immediately for the CAD-CAM (computer-aided design/computer-aided manufacturing) fabrication of the space maintainer. The appliance was delivered and cemented in the third appointment, ensuring timely intervention and preventing undesirable tooth movement.

Appliance Design

**Figure 4:**
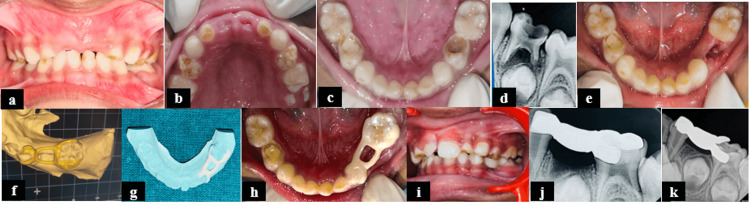
Ceramic band and loop space maintainer using CAD-CAM technology for Case 4. (a) Intraoral frontal view; (b) intraoral preoperative maxillary occlusal view; (c) intraoral preoperative mandibular occlusal view showing grossly decayed 74 and restored 84; (d) radiograph showing non-restorable carious teeth with respect to 74 with furcal involvement; (e) mandibular occlusal view after mouth preparation; (f) scanned impression with templates for the fabrication of ceramic band and loop space maintainer using CAD-CAM; (g) ceramic band and loop space maintainer using CAD-CAM; (h) mandibular occlusal view after space maintainer insertion; (i) right molar view after space maintainer insertion; (j) RVG of teeth 73, 74, and 75 region after placement of space maintainer; and (k) follow-up radiograph at six months. CAD-CAM: computer-aided design/computer-aided manufacturing

The appliance design process began with impression-making, where an alginate impression was taken, and the casts were poured using dental stone. In the laboratory procedure, the models were first digitalized using a digital scanner (Shining 3D Tech Co., Ltd., Wenyan Town, China), followed by manipulation of the digitalized data using Exocad 3.0 CAD software to design the template. The finalized design was then transferred to the CAM system, where milling facilitated the fabrication of the space maintainer. For appliance cementation, the abutment teeth 73 and 75, along with the inner surface of the ceramic space maintainer, were etched using 37% phosphoric acid (Neoetch Gel, Orikam Healthcare, Gurugram, India) and 5% hydrofluoric acid (Porcelain Etchant, Ultradent Products Inc., South Jordan, USA) for 20 seconds, respectively. Hydrofluoric acid was used for conditioning the inner surface of the ceramic space maintainer, in accordance with the manufacturer's instructions, to ensure optimal bonding. The teeth were then rinsed and air-dried for 10-15 seconds, followed by application of a bonding agent (Ivoclar Vivadent Te-Econom Bonding Agent), which was light-cured for 40 seconds. Finally, the ceramic band and loop space maintainer were cemented using resin-based luting cement (Luting 2 RMGIC, 3M™ RelyX™, St. Paul, USA) (Figure 4).

Case 5: Nikhil's appliance

Case Description

A six-year-old boy reported a missing tooth in the lower left back tooth region four months ago. The tooth was extracted owing to gross decay. Clinical examination revealed a prematurely missing 75 and a mesially tilted 36. RVG revealed a 3-4 mm amount of bone covering the developing premolar and mesially inclined 36. Tanaka and Johnston's space analysis was performed, wherein a space discrepancy was found to be 2.5 mm; hence, the use of a space regainer was indicated. A modified space maintainer, Nikhil's appliance, was planned to maintain the available space and reclaim the lost space simultaneously.

Appliance Design

**Figure 5:**
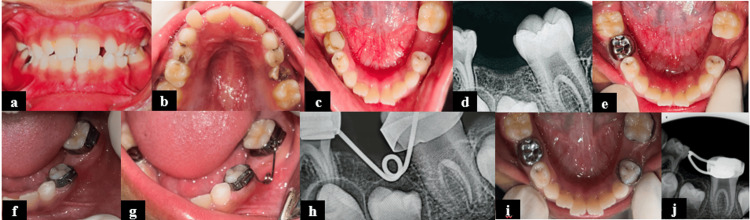
Nikhil's appliance for Case 5. (a) Intraoral frontal view; (b) intraoral preoperative maxillary occlusal view; (c) intraoral preoperative mandibular occlusal view showing prematurely missing 75 and mesially tilted 36; (d) radiograph showing un-erupted 35 with mesially tilted partially erupted 36; (e) mandibular occlusal view after mouth preparation; (f) banding with buccal tubes on teeth 36 and 74; (g) insertion of V-loop into buccal tubes; (h) radiograph after Nikhil's appliance insertion; (i) follow-up at two months; (j) radiograph after band and loop insertion.

Stainless-steel bands with buccal tubes (0.180 × 0.005) of appropriate size were adapted onto 36 and 74, followed by cementation using type-I GIC. A wire component made of 21-gauge (0.032 inches), having a V-loop with a helix at an angle of 30-45°, was incorporated into the buccal tubes after measuring the distance between two buccal tubes. The final adjustment is done by coiling/uncoiling the helix, and postoperative instructions were given. After two months, the patient came with a breakage of the appliance; hence, it was replaced with the band and loop using the same preformed band (Figure 5).

## Discussion

Various space maintainers can be used depending on the child's age, type of dentition, dental arch space, patient compliance, and needs. The band and loop appliance is the most commonly utilized space maintainer, indicated primarily for the premature loss of a single primary molar, whether unilateral or bilateral, in the maxillary or mandibular arch (Table 1).

**Table 1:** Indications of band and loop space maintainer

Indications of Band and Loop Space Maintainer
Preservation of space created by the premature loss of a single primary molar
Indicated by bilateral loss of primary molar teeth before the eruption of permanent incisors
Premature loss of primary canines
The root development of the un-erupted permanent premolar is less than one-third and has >2 years of eruption time

The fabrication of conventional band and loop space maintainers is tedious and technique-sensitive, with long chairside time demanding patience and cooperation from the patient (Table 2).

**Table 2:** Fabrication steps of conventional band and loop space maintainer

Steps	Procedure
Band adaptation	A 0.180×0.005-inch thickness of stainless-steel band material molded to form a ring was seated onto the tooth with a band pusher, pinched as close to the tooth surface as possible, and then spot welded. The band material was adapted along the tooth contour, followed by festooning and trimming.
Impression-making and band transfer	An alginate impression was made, and a band was transferred from the tooth to the desired position on the impression, which was stabilized using a tripod configuration.
Loop fabrication	The tooth to be extracted was scraped off the cast model. A loop was fabricated using 19-gauge (0.039 inches) wire with faciolingual dimensions of 8 mm.
Soldering	The band and the loop were soldered together using silver solder.

Hence, advancements in conventional methods have created various alternatives to mitigate the drawbacks associated with the conventional band and loop while retaining its positives [8]. Since the adaptation of the conventional band material is time-consuming, prefabricated bands in various sizes were introduced by RM Ricketts in 1935. They are indicated in cases of primary molar loss where quick, chairside delivery is needed, especially in uncooperative children. In a clinical trial, Setia et al. (2014) reported a high success rate of 92.3% with prefabricated bands and custom-made loops compared to 86.7% for the conventional types [9]. In the present case series, "prefabricated band and custom-made loop" space maintainers showed similar clinical performance in terms of survival rate as that of a conventional band and loop. However, these limitations remained the same, requiring at least two appointments with the need for impression- and technique-sensitive lab procedures. Another evolution to overcome these obstacles was the introduction of prefabricated band and loop space maintainers. Prefabricated space maintainers are easy to use and quick to adjust, which is especially beneficial for anxious young children requiring short appointments. Due to this, patients show high compliance and achieve better clinical performance at different follow-ups in Case 3. Dutta et al. reported a 100% survival rate for prefabricated space maintainers, compared to a 92% survival rate for conventional band and loop space maintainers [10].

The drawbacks of metal-based space maintainers include poor esthetics and potential metal allergies, leading to the development of metal-free space maintainers. GFRC space maintainers and CAD-CAM-assisted metal-free ceramic space maintainers are the most common types now used in pediatric dentistry. Bishara et al. reported that metal appliances corrode in the oral environment, releasing both nickel and chromium, risking allergic reactions [11,12]. The nickel release is higher in the autoclave-sterilized samples than in the non-sterilized samples [13]. Various GFRCs like Ribbond™, Fiber-Splint, and EverStick are used in dentistry for splinting, strengthening restorations, space maintainers, and bridge fabrication [14]. The GFRC space maintainers are more esthetic, less invasive, easy on soft tissue, easy postoperative removal, and highly accepted in pediatric patients. Tunc et al. (2012) reported a survival rate of 40% with a survival period of 6.7 months compared to a 100% survival rate of conventional band and loop at 11.2 months [15].

In the present case series, the GFRC space maintainer failed due to debonding at the enamel-composite interface at the one-month follow-up. Therefore, only the lingual strip of the GFRC was removed, while the buccal strip, which remained intact and did not interfere functionally or esthetically, was left in place (Figure 3j). The assessment was done using the criteria given by Kirzioglu [6], which were intended to evaluate the clinical performance and retention of the GFRC space maintainer over time. The criteria assessed include debonding at both the enamel-composite and fiber-composite interfaces, which indirectly reflects the bonding capacity and retention effectiveness of the bonding agent used [16].

Recently, CAD-CAM technology has achieved significant success in pediatric dentistry. The two main advantages of this approach are higher patient acceptance and better compliance. Khanna et al. (2021) found that space maintainers developed using CAD-CAM demonstrated excellent results, while the conventional ones showed depression and microfractures of the appliance due to occlusal forces [17]. In the present study, the highest success rate was observed for the metal-free ceramic space maintainer in terms of gingival inflammation, caries development, failure due to debonding, and difficulty in mastication. The appliance was well tolerated by the patient and had the advantages of advanced esthetics with strength and durability. However, it also presented challenges such as high costs, the need for specialized training, ongoing equipment maintenance, and software limitations. Hence, balancing these disadvantages with the benefits is essential for routine dental practices.

Nikhil's appliance, or the "tube and loop" appliance, is another innovative alternative introduced by Nikhil (2021) [18]. It only requires a single visit, eliminating the need for laboratory work. Its flexibility aids in space regaining, which is crucial for realigning and maintaining the space effectively. Tyagi et al. (2021) reported a 100% success rate in terms of survival time until the nine-month follow-up [19]. In the present case series, the patient came with appliance breakage after two months (Figure 5i). Hence, it was replaced with the band and loop using the same preformed band (Figure 5j).

In the present case series, RMGIC was used for luting the ceramic space maintainer, while GIC was used for the others. However, both showed similar responses in terms of retention. Longevity of the cement could not be determined after six months of follow-up. However, according to a recent study by Kaur et al., RMGIC provides superior retention and longer durability compared to conventional GIC [20].

Clinical significance

This case series highlights the importance of individualized treatment planning in pediatric patients, where space maintainers play a critical role in preserving dental arch space. The use of various modified space maintainers addresses diverse patient needs, optimizing esthetic outcomes, comfort, and compliance. Preformed bands and loops significantly reduce chairside time and improve patient compliance toward treatment. Esthetic space maintainers (GFRC, CAD-CAM-based) significantly improve appearance. Nikhil's appliance serves a dual purpose in terms of space maintenance and regaining.

## Conclusions

All the modifications of band and loop space maintainers demonstrated comparable success rates, making them viable alternatives in clinical practice. Dental professionals must weigh the distinct advantages and disadvantages of each type against the child's behavior, individual needs, potential benefits, and parental preferences. By thoughtfully assessing these factors, dental professionals can select the most suitable option, ensuring effective treatment that promotes optimal dental development and enhances patient outcomes.
